# A Multicentre Study of Psychological Variables and the Prevalence of Burnout among Primary Health Care Nurses

**DOI:** 10.3390/ijerph16183242

**Published:** 2019-09-04

**Authors:** Elena Ortega-Campos, Guillermo A. Cañadas-De la Fuente, Luis Albendín-García, José L. Gómez-Urquiza, Carolina Monsalve-Reyes, E. Inmaculada de la Fuente-Solana

**Affiliations:** 1 Faculty of Psychology, University of Almería. Carretera de Sacramento s/n, 04120 Almería, Spain; 2 Faculty of Health Sciences, University of Granada, Avenida de la Ilustración, 18016 Granada, Spain (G.A.C.-D.l.F.) (J.L.G.-U.); 3 Departamento de Ciencias Sociales, Universidad Católica de La Santísima Concepción, Avenida Alonso de Ribera, 2850 Concepción, Chile; 4 Brain, Mind and Behaviour Research Center (CIMCYC), University of Granada, Campus Universitario de Cartuja, 18071 Granada, Spain

**Keywords:** anxiety, burnout, depression, nursing, occupational health, personality factors, public health service

## Abstract

Nurses in primary health care (PHC) have multiple responsibilities but must often work with limited resources. The study’s aim was to estimate burnout levels among PHC nurses. A Quantitative, observational, cross-sectional, multicentre study of 338 nurses working in PHC in the Andalusian Public Health Service (Spain) is presented. A total of 40.24% of the nurses studied had high levels of burnout. The dimensions of emotional exhaustion and depersonalisation were significantly associated with anxiety, depression, neuroticism, on-call duty and seniority-profession and inversely related to agreeableness. In addition, depersonalisation was significantly associated with gender, and emotional exhaustion correlated inversely with age. Personal achievement was inversely associated with anxiety and depression and positively correlated with agreeableness, extraversion and responsibility. There is a high prevalence of burnout among nurses in PHC. Those most likely to suffer burnout syndrome are relatively young, suffer from anxiety and depression and present high scores for neuroticism and low ones for agreeableness, responsibility and extraversion.

## 1. Introduction

Primary health care (PHC) can be defined as basic, essential health care that is accessible to all members of a community. The cost of the service must be affordable to the participants, the community and the country. These considerations were highlighted at an international conference in this respect held in Alma-Ata in September 1978, where the urgent need was expressed for all governments and personnel involved in the development and promotion of health care to work for its universal availability [1]. According to a recent study, countries which emphasise the provision of PHC are better able to achieve their sustainable development goals and to promote the fairness and social justice on which the provision of universal health care is based [2].

Maslach and Jackson [3] provided the most widely accepted definition of burnout, describing it as a three-dimensional syndrome in which a worker experiences emotional exhaustion (EE), or feelings of physical overexertion and emotional fatigue as a result of the continuous interactions required between the worker and users of the service; depersonalisation (D) or the existence of cynical attitudes and responses towards the persons to whom services are provided; and a sensation of low personal achievement (PA) or the loss of confidence and the appearance of a negative self-concept due to encounters with unrewarding situation [4]. Among the various instruments proposed for assessing burnout, one of the most commonly used is the Maslach Burnout Inventory (MBI) [3]. Those affected by burnout usually work in contact with the public, as is the case of the health professions, and within this group, nurses are especially prone to burnout [5]. This syndrome not only impacts on workers but can also reduce the quality of care provided [6,7]. In recent years, the question of burnout syndrome has been addressed in literature reviews [8,9,10,11] and empirical studies [4,5], and work continues in this field, highlighting its importance in society [12].

In this context, an important consideration is that hospital nurses work in a specific clinical environment. Thus, the tasks performed regularly by nurses working in A & E, oncology or paediatrics departments, for example, differ greatly from those carried out in PHC [9,13,14,15].

The impact of burnout syndrome is normally analysed by reference to the different variables related to its presence. These may be sociodemographic (age, sex, marital status, number of children, etc.), employment-related (the hospital service in question, seniority in the workplace, seniority in the profession, etc.) or psychological (anxiety, stress, depression, personality traits, etc.). Identification of the variables/factors that influence the development of burnout syndrome would contribute to determining risk profiles for the nursing profession [16].

Little empirical research has been conducted into burnout syndrome in PHC nurses, in comparison with other hospital settings [17,18]. Further investigation in this respect, therefore, would help determine the prevalence of burnout in PHC nurses and facilitate its prevention [19].

This study has the following aims: (1) to estimate the level of burnout among nurses working in PHC in the Andalusian Public Health Service; (2) to determine the phases of burnout that most affect these nurses; (3) to analyse the relationship between sociodemographic, employment-related and personality variables and the burnout syndrome suffered by this group of persons.

## 2. Materials and Methods

### 2.1. Participants

The study sample was composed of 338 PHC nurses working in the Andalusian Public Health Service (Spain). These nurses were specialists or generalists who worked in community health centers, serving the entire community. The participants’ average age was 45.92 years (SD = 7.51), and 58% were female. All had university studies in nursing and were employed in this capacity. The response rate was 84.5%.

### 2.2. Variables, Instruments and Data Collection

A research questionnaire was used to record the data compiled. The sociodemographic variables studied were gender (male-female), age (years), marital status (single, married, separated/divorced, widowed) and number of children (none, one, two, three or more). The employment-related variables considered were work pattern (fixed or rotating shifts), on-call obligation (yes-no); seniority in the workplace (months) and seniority in the profession (months).

Burnout syndrome was measured using the MBI [3] in a version adapted to a Spanish-speaking population [20]. The instrument consisted of 22 items, scored on a seven-point Likert-scale, from zero (never) to six (every day), with respect to three dimensions: EE (nine items), D (five items) and PA (eight items). Cut-off points for the presence of burnout were defined by reference to established values for the Spanish population, namely, EE > 24, D > 9 and PA < 33. The estimated coefficients of reliability for these MBI scales were EE (α = 0.91), D (α = 0.72) and PA (α = 0.86).

The revised NEO personality inventory (NEO-FFI) instrument [21], adapted for use with a Spanish-speaking population [22], was also used. This instrument is composed of five personality factors: neuroticism, agreeableness, responsibility, extraversion and openness. Twelve items were considered for each of these dimensions (total, 60 items), and scored on a five-point Likert scale. The following reliability coefficients were obtained for the NEO-FFI dimensions: neuroticism (α = 0.83), agreeableness (α = 0.80), responsibility (α = 0.84), extraversion (α = 0.82) and openness (α = 0.67).

The Educational-Clinical Questionnaire on Anxiety and Depression (CECAD), proposed by Lozano, García-Cueto and Lozano [23], was used to measure symptoms of anxiety and depression, in accordance with the DSM-IV criteria. This questionnaire consists of 45 items scored on a five-point Likert scale, with 19 items for anxiety and 26 for depression. The following reliability coefficients were obtained for the CECAD dimensions: anxiety (α = 0.94) and depression (α = 0.96).

To obtain the necessary data, the investigators first contacted the Spanish Nursing Union (SATSE), whose staff then worked in conjunction with the research team to inform PHC nurses in the Andalusian Public Health System about the study and to request their participation, which was totally voluntary, individual and anonymous. It was estimated that each nurse would require 45 min to complete the study questionnaire. The data were compiled from January to June 2017. The study was approved by the Ethics Committee of the University of Granada (393/CEIH2017) and was conducted in full compliance with the ethical considerations of the Declaration of Helsinki (2008). All study data were treated in accordance with the relevant Spanish legislation on data protection (LOPD, 1999) [24].

### 2.3. Study Design

This study is quantitative, observational, cross-sectional and multicentre.

### 2.4. Data Analysis

Descriptive statistics (mean, standard deviation, minimum and maximum) were calculated for the quantitative variables, and frequencies and percentages for the qualitative ones. Hypothesis tests for independent groups were conducted to determine differences in MBI dimensions and in the qualitative variables. Pearson correlation coefficients were estimated for the quantitative variables. All analyses were performed using SPSS 23.0 statistical software (IBM, Armonk, NY, USA).

## 3. Results

### 3.1. Data, Levels of Burnout and Prevalence

Table 1 shows the percentages and frequencies obtained for the qualitative variables included in the study. Regarding the participants, 58% were women, 78.6% were married and 84.6% had at least one child. With respect to employment patterns, 76.6% worked a fixed shift (morning, afternoon or evening) and 86.8% performed on-call duties.

Table 2 presents the descriptive statistics (mean, standard deviation, minimum and maximum) obtained for the quantitative variables. Among the data for employment-related variables, the nurses had been employed for an average of 127.67 months (SD = 99.84) in the position and had an average of 273.66 months (SD = 91.38) of professional seniority as nurses. The following average scores were obtained for the NEO-FFI subscales: neuroticism 27.96 (SD = 8.33); extraversion 42.19 (SD = 7.90); openness 38.84 (SD = 6.68); agreeableness 44.80 (SD = 7.54); responsibility 46.73 (SD = 7.37). In the ECCAD dimensions, the mean scores obtained were 36.43 (SD = 11.66) for anxiety and 50.05 (SD = 17.03) for depression. Finally, the mean MBI scores were 17.70 (SD = 11.92) for EE, 7.59 (SD = 6.25) for D and 36.59 (SD = 8.90) for PA.

In relation to the EE dimension of burnout, 48.1% of the nurses presented a low level, 25.4% a moderate level and 26.6% a high level. For D, the corresponding figures were 35.1%, 27.6% and 37.2%, while for PA they were 43%, 28.7% and 28.4%.

Following the model proposed by Golembiewski, Munzenrider and Carter [25], the nurses were classified according to the stage of evolution of burnout syndrome. Phases 6, 7 and 8 of this model reflect a high level of burnout. According to this parameter, 40.24% (*n* = 134) of the nurses in our sample presented a high level of burnout (Table 3).

### 3.2. Exploratory Models and Factors Associated with Each of the Dimensions of Burnout

The linear correlation coefficients between the MBI scales, the NEO-FFI scales and the anxiety and depression scores were also calculated. The EE scale presented the following statistically significant correlations with the NEO-FFI scores: neuroticism (*r* = 0.62), extraversion (*r* = −0.43), agreeableness (*r* = −0.45) and responsibility (*r* = −0.42). Significant correlations were also obtained with the CECAD scores for anxiety and depression (*r* = 0.67 and *r* = 0.69, respectively). On the D scale, statistically significant relationships were found with the following NEO-FFI and CECAD scores: neuroticism (*r* = 0.48), extraversion (*r* = −0.38), openness (*r* = −0.21), agreeableness (*r* = −0.49), responsibility (*r* = −0.44), anxiety (*r* = 0.47) and depression (*r* = 0.48). Finally, statistically significant correlations were found between the PA scale and the following NEO-FFI and CECAD scores: neuroticism (*r* = −0.42), extraversion (*r* = 0.49), openness (*r* = 0.25), agreeableness (*r* = 0.49), responsibility (*r* = 0.55), anxiety (*r* = −0.40) and depression (*r* = −0.45) (Table 4).

The difference of the means test was conducted for the variables gender, shift work, on-call duties, marital status and children, against the MBI dimensions. Statistically significant differences were found in the D scale between men and women (*t* (_331_) = 2.52, *p* = 0.012, *d* = 0.28), with men presenting higher levels. With respect to on-call duties, statistically significant differences were found in the EE scale (*t* (_232,646_) = 1.98, *p* = 0.048, *d* = 0.23) and D (*t* (_330_) = 2.85, *p* = 0.005, *d* = 0.31), with higher scores recorded for EE and D by the nurses who performed on-call duties.

Multiple linear regression models were estimated for each of the MBI dimensions. For the EE dimension, the variables age (B = −0.31, *p* = 0.024), seniority in the profession (B = 0.02, *p* = 0.046), neuroticism (B = 0.32, *p* ≤ 0.001), agreeableness (B = −0.17, *p* = 0.013), anxiety (B = 0.30, *p* = 0.001) and depression (B = 0.14, *p* = 0.035) were statistically significant predictors. For goodness of fit, the model presented a value R^2^ = 0.553, meaning that 55.3% of the variability of EE was explained by the model.

With respect to the D dimension, the variables age (B = −0.09, *p* = 0.027), seniority in the position (B = 0.01, *p* = 0.027), neuroticism (B = 0.12, *p* = 0.013), agreeableness (B = −0.26, *p* ≤ 0.001) and anxiety (B = 0.11, *p* = 0.001) were predictors in the model. The model explained 35.4% of the variance of the variable D.

Finally, in the PA dimension, the variables agreeableness (B = 0.19, *p* = 0.004), responsibility (B = 0.38, *p* ≤ 0.001), extraversion (B = 0.22, *p* ≤ 0.001) and depression (B = −0.06, *p* = 0.030) were predictors. 40.2% of the variability in PA was explained by the model (Table 5).

## 4. Discussion

PHC is considered less stressful than other hospital services, with patients who are more stable and present fewer complications [19]. In our study sample, although the percentage of male nurses was higher than in other studies, the group was sufficiently representative of the PHC nursing population. In terms of employment patterns, these nurses had considerable responsibilities, with most working a fixed shift (either mornings or afternoon/evenings) and, in addition, subject to on-call obligations ranging from 17 to 24 h per day. As a result, the work overload was significant, an adverse condition that contributed to the high levels of burnout prevalence observed among this population [26].

EE is the most representative dimension of burnout. This outcome becomes more acute with greater age and seniority in the position, due to the stress and monotony encountered and/or job dissatisfaction [19,27]. In addition, in our study sample, a significant proportion of the nurses were married. This factor, representing additional family commitments, also increases EE [27,28]. Our analyses highlight the existence of an important relationship between EE, anxiety, depression and personality traits. The skills required of the nurses and their heavy workload (especially as concerns on-call duties) are directly related to the dimension of responsibility [29,30]. The resulting stress and anxiety, moreover, contribute to feelings of neuroticism [26,31], such that some nurses consider abandoning the profession and, in the most extreme cases, may become suicidal [19]. In this respect, openness and extraversion are protective factors, mediating in stress and work-related problems [31,32].

Depression is another common manifestation of burnout among nurses. When levels of PA are high (for instance, due to the empathy and accompaniment implicit in caring for chronic patients), D is not usually very severe [30] but as with EE, it increases with age and the resulting lack of motivation [27] and this, in turn, decreases extraversion [31]. When nurses are subjected to heavy workloads, with an inefficient distribution of tasks and insufficient resources, they may view the environment as hostile [17,33] and experience anxiety, feelings of vulnerability, stress, lack of responsibility and depression, all of which increase the sensation of burnout [11,19,26,29,31,32,34]. Furthermore, agreeableness and openness—protective factors that would otherwise contribute to averting burnout– are both adversely affected [31,32].

In PHC, although nursing personnel may have considerable job satisfaction, it is not uncommon to find low levels of PA, since the degree of responsibility is high [29]. Nursing is a highly vocational profession [35] and so agreeableness plays an important role in this dimension, and this helps moderate the stress experienced [32]. On the other hand, some aspects of nursing work are viewed very negatively, especially the obligation to perform on-call duties. Not all nurses are psychologically or physically prepared for this obligation, which can generate stress, anxiety and lack of adaptation [26,31], frustration and work overload [30] and, sometimes, depression [19]. Adverse employment conditions can also cause extraversion and openness to be diminished, reducing nurses’ ability to cope with problems that may arise [31].

In view of the results obtained, risk profiles for PHC nurses can be established. Persons who present high scores for EE and D and low ones for PA tend to be younger and to suffer from anxiety and depression. Moreover, their limited experience and exposure to organisational problems and even a hostile work environment can provoke negative attitudes [11,19,36,37].

With respect to personality factors, the results show that PHC nurses who obtain high scores for neuroticism and low ones for agreeableness, responsibility and extraversion are also at risk of presenting burnout syndrome. When a nurse has a high level of neuroticism, this is usually associated with an increased severity and frequency of personal problems [38]. Such an individual will often form part of the hierarchical structure of the organisation but not involve themselves in the job and will frequently be dissatisfied with it [5]. Low levels of agreeableness, responsibility and extraversion often mean less involvement and a decreased quality of care [5], poorer social skills [38] or unsatisfactory work conditions [11,26,29,30], all of which can result in reduced self-efficacy, increased hostility and a bad nurse–patient relationship [4]. These outcomes are consistent with the findings of previous studies, according to which the greater the number of negative personality traits in a nurse, the greater their risk of suffering from burnout [38].

One of the objectives of the study was to determine the burnout phase in which the nurses are. According to the data, the group of nurses surveyed present high levels of burnout. Based on Golembiewski’s model [25] 40% are in the highest stages of burnout. These results are in line with recent studies [4,13,19]. The Golembiewski classification [25] is not widely used in studies on burnout, it would be advisable to include it because it provides important information especially regarding the classification and diagnosis of burnout syndrome.

The prevention of burnout helps create a positive working environment, promotes the autonomy of nurses and facilitates achievement of their objectives [37]. Psychological and organisational interventions in this respect [31], especially for younger and less experienced staff [36,38] would significantly contribute to reducing burnout among PHC nurses. Such interventions might include mindfulness programmes, the provision of social support and/or the development of protocols against violence in the workplace [17,33,34,39,40]. These initiatives could enhance personal protective factors, such as extraversion, agreeableness and openness [19,32,38] and organisational ones such as teamwork and job satisfaction, in an environment that can be very demanding and where resources are often insufficient [11,29].

Future research should analyse the influence of these burnout risk factors through longitudinal studies to establish causality relationships. Other variables, such as biochemical factors that may help the clinical diagnosis of burnout, should also be taken into account. Finally, it would be interesting to analyse the effect of some interventions on the reduction of burnout in primary care nurses.

## 5. Conclusions

The prevalence of burnout is high among PHC nurses, especially those who are younger, suffer from anxiety and depression and present high levels of neuroticism and low ones of agreeableness, responsibility and extraversion. This profile of PHC nurses that may be more prone to burnout syndrome should be taken into account by healthcare managers to establish preventive measures. The implementation of measures to improve the working conditions of PHC nurses would reduce levels of burnout and, therefore, enhance the quality of care provided.

## Figures and Tables

**Table 1:** Descriptive data for the qualitative study variables.

Variable	% (*n*)	Variable	%(*n*)
Gender (*n* = 338)		Marital status (*n* = 336)	
Male	42 (142)	Single	14 (47)
Female	58 (196)	Married	78.6 (264)
Shift (*n* = 334)		Separated/Divorced	6.3 (21)
Fixed	76.6 (256)	Widower	0.9 (3)
Variable	23.4 (78)	Children (*n* = 332)	
On-call (*n* = 337)		None	15.4 (51)
Yes	68.8 (232)	One	19 (63)
No	31.2 (105)	Two	45.5 (151)
		Three or more	20.1 (67)

**Table 2:** Descriptive data for the quantitative study variables.

Variable	Mean (SD)	Min-Max
Age (*n* = 337)	45.92 (7.510)	25–64
Seniority: Workplace (*n* = 326)	127.67 (99.84)	1–444
Seniority: Profession (*n* = 338)	273.66 (91.38)	12–504
NEO-FFI		
Neuroticism (*n* = 332)	27.96 (8.33)	12–53
Extraversion (*n* = 336)	42.19 (7.90)	20–60
Openness (*n* = 333)	38.84 (6.68)	20–57
Agreeableness (*n* = 330)	44.80 (7.54)	19–60
Conscientiousness (*n* = 334)	46.73 (7.37)	20–60
CECAD		
Anxiety (*n* = 334)	36.43 (11.66)	19–69
Depression (*n* = 329)	50.05 (17.03)	26–102
MBI		
Emotional exhaustion (*n* = 335)	17.70 (11.92)	0–54
Depersonalisation (*n* = 335)	7.59 (6.25)	0–29
Personal Accomplishment (*n* = 335)	36.59 (8.90)	2–48

CECAD = Educational-Clinical Questionnaire on Anxiety and Depression; MBI = Maslach Burnout Inventory; NEO-FFI = Revised NEO Five Factor Inventory; SD = Standard deviation; Seniority = presented in months.

**Table 3:** Prevalence of burnout according to the stages of the Golembiewski model.

Phase	1	2	3	4	5	6	7	8
EE	L	L	L	L	H	H	H	H
D	L	H	L	H	L	H	L	H
PA	L	L	H	H	L	L	H	H
*n*	41	26	89	24	19	86	20	28
(%)	(12.31)	(7.81)	(26.73)	(7.21)	(5.7)	(25.83)	(6)	(8.41)

H = High; L = Low; EE = Emotional exhaustion; D = Depersonalisation; PA = Personal accomplishment.

**Table 4:** Correlation coefficients between psychological variables and Burnout.

PV	EE	D	PA
**NEO-FFI**			
Neuroticism	0.62 **	0.48 **	−0.42 **
Extraversion	−0.43 **	−0.38 **	0.49 **
Openness	−0.08	−0.21 **	0.25 **
Agreeableness	−0.45 **	−0.49 **	0.49 **
Conscientiousness	−0.42 **	−0.44 **	0.55 **
**CECAD**			
Anxiety	0.67 **	0.47 **	−0.40 **
Depression	0.69 **	0.48 **	−0.45 **

PV = Psychological variables; EE = Emotional exhaustion; D = Depersonalisation; PA = Personal accomplishment; CECAD = Educational-Clinical Questionnaire on Anxiety and Depression; NEO-FFI = Revised NEO Five Factor Inventory; ** = *p* < 0.001.

**Table 5:** Multiple linear regression.

Regression	B	Standard Error	Beta	*t*	*p*	CI 95%
**EE**						
Intercept	6.16	5.82		1.05	0.291	(−5.29, 17.62)
Age	−0.31	0.13	−0.19	−2.26	0.024	(−0.58, −0.04)
Seniority: Profession	0.02	0.01	0.17	2.00	0.046	(0.00, 0.04)
Neuroticism	0.32	0.07	0.23	4.25	<0.001	(0.17, 0.47)
Agreeableness	−0.17	0.07	−0.11	−2.48	0.013	(−0.31, −0.03)
Anxiety	0.30	0.09	0.30	3.22	0.001	(0.12, 0.49)
Depression	0.14	0.06	0.20	2.11	0.035	(0.01, 0.27)
R^2^ = 0.553						
**D**						
Intercept	15.40	3.34		4.60	<0.001	(8.83, 21.98)
Age	−0.09	0.04	−0.11	−2.22	0.027	(−0.17, −0.01)
Seniority: Workplace	0.01	0.00	0.11	2.22	0.027	(0.00, 0.01)
Neuroticism	0.12	0.04	0.16	2.48	0.013	(0.02, 0.21)
Agreeableness	−0.26	0.04	−0.31	−5.71	<0.001	(−0.35, −0.17)
Anxiety	0.11	0.03	0.20	3.31	0.001	(0.04, 0.17)
R^2^ = 0.354						
**PA**						
Intercept	3.76	4.49		0.83	0.402	(−5.07, 12.60)
Agreeableness	0.19	0.06	0.16	2.91	0.004	(0.06, 0.32)
Conscientiousness	0.38	0.07	0.31	5.46	<0.001	(0.24, 0.52)
Extraversion	0.22	0.06	0.19	3.58	<0.001	(0.10, 0.34)
Depression	−0.06	0.02	−0.11	−2.17	0.030	(−0.11, −0.01)
R^2^ = 0.402						

B = Estimated parameter; CI = Confidence interval; EE = Emotional exhaustion; D = Depersonalisation; PA = Personal accomplishment.
